# Neuronal regeneration after injury: a new perspective on gene therapy

**DOI:** 10.3389/fnins.2023.1181816

**Published:** 2023-04-21

**Authors:** Chih-Wei Zeng, Chun-Li Zhang

**Affiliations:** ^1^Department of Molecular Biology, University of Texas Southwestern Medical Center, Dallas, TX, United States; ^2^Hamon Center for Regenerative Science and Medicine, University of Texas Southwestern Medical Center, Dallas, TX, United States

**Keywords:** neuronal regeneration, spinal cord injury, gene therapy, reprogramming, cell differentiation

## Introduction

Spinal cord injury (SCI) is a major cause of disability worldwide and regenerative medicine offers hope for the development of new therapies for these injuries (James et al., 2019). SCI can result in the loss of sensory and motor function and can have a profound impact on an individual's quality of life, affecting not only physical abilities but also emotional and social wellbeing (Eckert and Martin, 2017). Despite decades of research, there is still no cure for SCI. The lack of regeneration of injured neurons in the spinal cord is one of the major challenges in the field of regenerative medicine. In mammal, the spinal cord is a complex structure with limited capacity for regeneration (He and Jin, 2016; Sofroniew, 2018), and the cellular and molecular mechanisms that regulate neuronal regeneration are not fully understood.

Recent studies have identified new targets and potential strategies for promoting neuronal regeneration, including the use of stem cell therapy (Okano, 2010; Führmann et al., 2017), gene therapy (Lentini et al., 2021; Zhang Y. et al., 2022), and tissue engineering (Madhusudanan et al., 2020; Cheng et al., 2021). The use of gene therapy in promoting regeneration and functional recovery in various conditions has been highlighted in recent studies. For instance, gene therapy using a time-restricted glial cell line-derived neurotrophic factor expression via an immune-evasive doxycycline-inducible gene switch has shown promise in enhancing axon regeneration and motor neuron survival after proximal nerve lesions in rats (Eggers et al., 2019). Resident astrocytes were shown to generate new neurons after SOX2-mediated *in vivo* fate reprogramming (Su et al., 2014; Wang et al., 2016). Similarly, another study revealed that ectopic SOX2 in NG2 glial cells can induce neurogenesis, reduce glial scarring, and generate propriospinal neurons, promoting functional recovery (Tai et al., 2021). Moreover, the delivery of FGF22 gene therapy after spinal cord injury has been shown to promote synaptogenesis and targeted support for neuronal rewiring, with acute and early application improving functional recovery (Aljović et al., 2023). However, the results reveal the presence of a narrow time frame, at least within the first 24 h after SCI, during which synaptogenic gene therapy with FGF22 can improve recovery of motor function. This limited window might be difficult to achieve in a clinical setting, which may necessitate the exploration of alternative synaptogenic molecules or approaches with a more extended therapeutic window. Overall, these findings suggest that gene therapy has the potential to activate the regenerative ability of endogenous glial cells, leading to regeneration and functional recovery in various conditions.

Gene editing is another type of gene therapy (Boulad et al., 2018) that shows promise in promoting regeneration in various neurodegenerative disease models. Techniques such as CRISPR-Cas9 and iPSCs can correct genetic mutations that contribute to regeneration or reduce the risk of developing neurodegenerative diseases like Parkinson's disease (Chavez et al., 2023) and Alzheimer's disease (György et al., 2018). For instance, CRISPR/Cas9 can disrupt mutations in genes like *APPSwe*, which reduces the secretion of Aβ and may be used for gene therapy against Alzheimer's disease. Therefore, gene editing has the potential to correct genetic mutations, promote regeneration, and reduce the risk of neurodegenerative diseases.

Despite advancements in developing therapies for SCI, significant challenges related to safety, efficacy, and scalability remain. Furthermore, a comprehensive understanding of the mechanisms underlying neuronal regeneration is lacking, hindering the development of effective therapies. Gene editing, which allows for precise modification of genetic sequences, offers a promising avenue for the correction of disease-causing mutations or engineering of cells to promote tissue repair and regeneration. While stem cell technology has shown promise in animal models of SCI, caution is warranted due to limitations in translating these findings to human patients. Additionally, while mesenchymal stem cells may exhibit neuron-like characteristics, their efficacy seems primarily related to their paracrine activity rather than cellular replacement mechanisms.

In this opinion article, we present a viewpoint on the potential of gene therapy in regenerative medicine for treating SCI and discuss its limitations and future directions. We also discuss the importance of comparative studies of neuronal regeneration in different vertebrate species and the potential of combining different approaches, including gene therapy, to promote neuronal regeneration in the spinal cord. By better understanding the mechanisms that regulate neuronal regeneration and developing safe and effective therapies, we can improve the outcomes for patients with SCI and other neurological disorders.

## Current landscape and challenges

### Importance of comparative studies of neuronal regeneration in the spinal cord

The study of neuronal regeneration in different vertebrate species, such as zebrafish and axolotls, has allowed for a better understanding of the mechanisms of regeneration and the identification of factors that influence regeneration. For example, zebrafish have a high potential for neuronal regeneration (Zeng et al., 2016, 2020; Lee et al., 2022). Recent studies have identified potential candidate genes that can induce neuronal regeneration after SCI, such as connective tissue growth factor a (CTGFa) (Mokalled et al., 2016). This extracellular matrix (ECM) protein can improve spinal cord repair in injured zebrafish. Additionally, Caveolin 1 (Cav1), a membrane protein, was significantly upregulated in the rostral side of glial cells at the injury region and was found to be responsible for axonal regrowth (Zeng et al., 2021). It is noteworthy that Cav1 is required not only for CTGF upregulation in the mesangial cells of the kidney (Guan et al., 2013), but also for high CTGF expression in hepatocytes (Pavlides et al., 2010). This evidence demonstrates that the presence of Cav1 can impact CTGF expression in kidney mesangial cells, and hepatocytes. Furthermore, H_2_O_2_ and its downstream effector CTGF are pro-regenerative factors that enable axonal growth and reveal a striking ECM remodeling process during nerve regeneration in a mouse model (Negro et al., 2022).

The above evidence suggests that comparative studies have identified signaling pathways and molecules involved in the process of regeneration and have provided insights into how these pathways can be targeted to promote regeneration in mammals. Therefore, the comparison of regenerative and non-regenerative species has highlighted the importance of the cell response and glial cells in promoting regeneration.

### Limitations and challenges of current strategies for promoting neuronal regeneration

Current strategies for promoting neuronal regeneration in the spinal cord, such as stem cell therapy and gene therapy, face significant limitations and challenges. Stem cell therapy has shown some promise in pre-clinical studies, but its clinical application is hindered by concerns about the potential for tumorigenesis, limited cell survival, and the lack of integration of transplanted cells into the host tissue. For example, in the case of Parkinson's disease evidence suggests that only 3–20% of grafted dopaminergic cells survive after the procedure (Brundin et al., 2000; Kim et al., 2020; Hiller et al., 2022). The loss of dopaminergic cells in grafts can be triggered by various factors, such as mechanical trauma, neuroinflammation, poor vascularization, and growth factor deprivation in the host brain (Brundin et al., 2000; Moriarty and Dowd, 2018). The critical period during which most dopaminergic cells die is the first few days after transplantation (Sortwell et al., 2000). To address these challenges, utilizing cells with neurotrophic properties to modify the pathologic brain environment or promote neuroprotective effects has emerged as a potential strategy for central nervous system (CNS) disorders. Despite advances in SCI research, clinical therapeutic approaches that promote neurological recovery are still limited (Elizei and Kwon, 2017). Currently, the clinically applicable methods for patients with SCI within the first 48 h mainly include spine immobilization (Ottosen et al., 2019) and surgical decompression (Badhiwala et al., 2021). Chemical treatments for SCI are designed to reduce inflammation, prevent further damage, and promote healing and recovery. While these treatments have shown potential in improving sensorimotor recovery in a treatment time-related manner, high-dose chemical treatment may increase the risk of adverse events in patients with acute SCI, with minimal contribution to neurological recovery. It is important to note that the effectiveness and safety of these treatments may vary, and more research is needed to fully understand their potential for treating SCI. Here are a few examples of chemical treatments for SCI: (1) Corticosteroids, such as methylprednisolone (MP): MP has been used to treat acute SCI due to its anti-inflammatory and neuroprotective effects (Bydon et al., 2014); (2) Riluzole: This drug has been shown to have neuroprotective properties by inhibiting the release of glutamate, a neurotransmitter that can cause excitotoxicity and contribute to secondary injury after SCI (Nagoshi et al., 2015); and (3) Glibenclamide: Glibenclamide targets SUR1-TRPM4 ion channels, which are upregulated after SCI. Blocking these channels has been shown to reduce edema and improve functional recovery in preclinical models (Kurland et al., 2013).

In the past, significant progress has been made in understanding the mechanisms that limit therapeutic outcomes after SCI. These mechanisms can be classified as extrinsic or intrinsic to the neurons. Efforts have been focused on investigating inhibitory factors related to glial scars (Sami et al., 2020), myelin debris (Li et al., 2020), and axonal components (Yang et al., 2020; Cheng et al., 2021). For example, Nieuwenhuis et al. (2020) highlights the importance of PI3Kδ in regulating axon outgrowth and regeneration in the adult CNS. By enhancing axonal PIP3 levels, PI3Kδ supports the regenerative capacity of injured neurons, making it a potential therapeutic target for promoting neural repair after injury. However, achieving axon regeneration remains the first step in promoting functional recovery. Additionally, long-distance axon growth across injured sites and proper neuronal relays with targets have been difficult to achieve (Shen et al., 2022). Therefore, due to the complex mechanisms and unusual difficulty of neural regeneration in SCI, it is unlikely that a single method or strategy will achieve sufficient results to generate functional recovery. In addition, identifying presynaptic molecules that can be targeted to promote axon regeneration and recovery after injury is of significant importance in the field of neuroscience. Recent studies have shed light on the molecular mechanisms that inhibit axon regeneration in the adult CNS, suggesting potential therapeutic targets for neural repair. For instance, Hilton et al. (2022) explored the role of vesicle priming machinery in axon regeneration following CNS injury. Their study revealed that an active vesicle priming process, governed by a protein complex called Munc13-1/2, hinders axon regeneration in adult CNS neurons. By reducing the levels of Munc13-1/2, they found that axon regeneration in injured neurons was promoted. This indicates that the vesicle priming machinery plays a crucial role in suppressing axon regrowth and could be a potential target for promoting neural repair. Similarly, Tedeschi et al. (2016) discovered that the expression of the calcium channel subunit alpha2delta2 is upregulated in injured adult CNS neurons, leading to the suppression of axon regeneration. By decreasing the levels of alpha2delta2 in injured neurons, the researchers successfully promoted axon regrowth and functional recovery. In conclusion, future studies should continue to investigate the molecular mechanisms that inhibit axon regeneration in the adult CNS. This will enable researchers to identify novel therapeutic targets, such as the vesicle priming machinery and the calcium channel subunit alpha2delta2, which could be leveraged to promote neural repair after injury.

Taken together, there are various approaches attempting to increase functional recovery after SCI, but a satisfactory solution has not yet been found. In summary: (1) Stem cell therapy is the current strategy for promoting neuronal regeneration in the spinal cord, but it faces significant limitations and challenges, such as low cell survival rates, tumorigenesis, and lack of integration into host tissue; (2) Clinical therapeutic approaches that promote neurological recovery for SCI are still limited; (3) The complex mechanisms and unusual difficulty of neural regeneration in SCI make it unlikely that a single method or strategy will achieve sufficient results to generate functional recovery.

Continuous efforts are needed to explore new ways of generating functional recovery after SCI using current technology. While gene therapy for regeneration has been a hot topic in recent years, this technology is not yet well-suited for application in mammalian spinal cord regenerative models. In the next section, we propose a few different views of gene therapy that could offer a fresh perspective on regenerative medicine after SCI in mammalian.

### The potential of gene therapy in promoting neuronal regeneration

For over three decades, researchers have been investigating the potential of genetic modifications to provide long-lasting and potentially curative treatments for various inherited human diseases with a single intervention. In the context of SCI, gene therapy can be utilized to engineer cells that promote neuronal regeneration. This approach is typically employed post-injury or after a disease has manifested, aiming to enhance tissue repair and regeneration. For instance, Lu et al. (2012) showcased the ability of genetically modified neural stem cells, engineered to express a combination of growth factors, to support axonal regeneration and functional improvement following SCI. Similarly, Pearse et al. (2004) illustrated the use of gene therapy to overexpress the enzyme adenylyl cyclase in Schwann cells, which in turn elevated cyclic AMP (cAMP) levels and promoted axonal regeneration after SCI in rats. The authors found that the combined approach of gene therapy and Schwann cell transplantation resulted in enhanced functional recovery.

Above studies highlight the promising role of gene therapy in fostering neuronal regeneration and functional recovery in the context of SCI. Typically applied after the onset of injury or disease, gene therapy aims to facilitate tissue repair and regeneration. Our focus is on the treatment of SCI through the use of nanostructured biomaterials and *in vivo* gene expression reprogramming.

#### Nanostructured biomaterials based therapy

Nanoparticles have the potential to revolutionize the field of medicine by enabling targeted drug delivery and improving the effectiveness of therapies for a wide range of diseases, including SCI. Currently, there are limited treatment options available for patients with SCI, and most therapies focus on managing symptoms rather than promoting recovery. However, nanoparticle-based therapies hold promise for improving outcomes in patients with SCI.

Nanoparticles offer a promising approach for delivering drugs or other therapeutic agents in the treatment of SCI. One such example is the CAQK peptide, which has demonstrated potential in treating CNS injuries due to its selective binding to chondroitin sulfate proteoglycans, which increase in expression following CNS injury (Mann et al., 2016; Abi-Ghanem et al., 2022). Another promising example involves the use of curcumin-loaded nanoparticles for SCI treatment. Curcumin, a natural polyphenolic compound derived from the turmeric plant, exhibits anti-inflammatory, antioxidant, and neuroprotective properties (Vaiserman et al., 2020; Sabouni et al., 2023), making it a promising candidate for addressing SCI (Krupa et al., 2019). However, the therapeutic efficacy of curcumin is limited by its low bioavailability and poor water solubility. To overcome these challenges, Ayyanaar et al. (2019) developed poly(lactic-co-glycolic acid) (PLGA) nanoparticles loaded with curcumin to enable targeted delivery to the injured spinal cord. Their study demonstrated that curcumin-loaded PLGA nanoparticles efficiently targeted the injury site, reduced inflammation, protected neurons, and promoted functional recovery in a rat model of SCI. These results suggest that curcumin-loaded nanoparticles hold promise as a therapeutic strategy for SCI treatment.

Another approach to mitigating detrimental hyperexcitability and neurotransmitter imbalance following SCI involves targeting specific neurotransmitter receptors. For instance, inhibiting acetylcholine receptors, particularly α7 nicotinic acetylcholine receptors (α7 nAChRs), has been shown to improve neuropathic pain behaviors by decreasing dynorphin A release (Ji et al., 2019). Moreover, targeting ionotropic glutamate receptors, such as α-amino-3-hydroxy-5-methyl-4-isoxazolepropionic acid (AMPA) receptors, is another promising strategy for SCI treatment (Park et al., 2003). AMPA receptors mediate fast excitatory synaptic transmission in the CNS, and their antagonists can counteract excitotoxicity by blocking excessive receptor activation (Gwak et al., 2007). This ultimately protects neurons from glutamate-induced damage. For example, the AMPA receptor antagonist NBQX (2,3-dihydroxy-6-nitro-7-sulfamoyl-benzo[f]quinoxaline) has demonstrated reduced neuronal death, improved tissue preservation, and enhanced functional recovery in animal models of SCI (Schidlitzki et al., 2017; Zhang J. et al., 2022). To achieve effective dosing, it is crucial for nanoparticles carrying these therapeutic agents to accumulate at the injury site. Nanoparticle design plays a significant role in enhancing local accumulation, which addresses a major challenge in drug delivery to the CNS: minimizing accumulation outside of the injury site. In summary, strategies targeting neurotransmitter receptors, such as α7 nAChRs and AMPA receptors, along with carefully designed nanoparticles, can help reduce excitotoxicity, promote neuronal survival, and improve functional recovery after SCI.

Nanoparticles have multiple advantages as tissue engineering tools for various pathologies, effectively enhancing regenerative medicine strategies. Studies reveal that chondroitin sulfate proteoglycans (CSPGs) enrichment is contributed by most cellular components close to the lesion epicenter for several months after injury, with CSPGs being the primary components of neural ECM (Beller and Snow, 2014; Yang et al., 2020). However, the upregulation of CSPGs after SCI results in dense isolation of the injury site, hindering neural regeneration and leading to permanent deficits. Consequently, treatments targeting CSPGs are gaining importance, as reducing CSPGs has been shown to enhance neural regeneration in SCI. For instance, Cafferty et al. (2007) transplanted genetically modified astrocytes expressing chondroitinase ABC into injured spinal cords of mice. The study revealed that chondroitinase ABC facilitated axonal regeneration by degrading inhibitory CSPGs, ultimately improving functional recovery. Similarly, Alilain et al. (2011) examined the combined effects of chondroitinase ABC treatment and rehabilitation training in rats with cervical SCI, finding enhanced functional recovery and increased synaptic connectivity. Additionally, Prado et al. (2019) assessed chondroitinase ABC administration in dogs with naturally sustained SCI injuries, showing improved locomotor function in some treated animals, supporting its potential as a promising therapeutic option for SCI treatment in humans.

On the other hand, Zhang X. et al. (2022) showed that paclitaxel-encapsulated PCL@SAD nanoparticles, when administered at the injury site, counteract the inhibitory effects of CSPGs, promote neural regeneration, provide neuroprotection to the damaged spinal cord, and improve locomotor recovery. PCL@SAD is a composite biomaterial scaffold composed of encapsulated paclitaxel (PCL) and acetal dextran nanoparticles (SAD). PCL acts as a biodegradable and biocompatible polymer, offering structural support, while SAD forms a nanofiber hydrogel that mimics the natural ECM, supporting cell adhesion and growth (Bouissou et al., 2014; Turner et al., 2020). PCL@SAD scaffolds integrate PCL's structural integrity with SAD's biological properties, creating an optimal environment for neural tissue regeneration (Hellal et al., 2011). These scaffolds open a new perspective toward the application of dextran-based nanoparticles for the treatment of severe neurological diseases.

Future research should focus on developing novel nanoparticle formulations by exploring innovative nanoparticle designs, materials, and surface modifications; optimizing drug release kinetics and targeted delivery; enhancing biocompatibility and safety; and investigating the potential for combined therapies that synergistically promote functional recovery and regeneration after SCI.

#### *In vivo* gene expression reprogramming therapy

Gene therapy holds great promise in treating a variety of human diseases, including spinal cord injuries. The use of gene therapy in SCI involves introducing new genes into cells in the spinal cord to promote regeneration and repair of damaged tissue. The approach involves modifying cells to produce proteins that promote the growth and differentiation of neural cells.

Recent studies have demonstrated the crucial role of transcription factors (TFs) in regulating the proliferation and differentiation of neural progenitor cells into neurons during neurodevelopment. TFs are proteins that bind to DNA and control gene transcription. By reintroducing the expression of key TFs in glial cells in the spinal cord, researchers aim to reactivate neurogenic potential and promote neuroregeneration. SOX2 is a TF that maintains the identity of neural progenitor and stem cells. Recent studies have shown that SOX2 can reprogram astrocytes into proliferating neuroblasts, which can further differentiate into mature neurons with additional treatments. For example, Su et al. (2014) used a lentiviral vector to introduce the SOX2 TF into reactive astrocytes in the injured adult spinal cord. This approach enabled the conversion of reactive astrocytes into functional neurons in mice after spinal cord injury. In another study, Wang et al. (2016) employed a lentiviral vector to express SOX2 in spinal cord astrocytes. The vector was injected into the injured spinal cord of adult mice, allowing for the overexpression of SOX2 in astrocytes and resulting in the generation of induced neurons. However, it is important to note that the dynamic expression of SOX2 is essential during the reprogramming process. Persistent expression of SOX2 can inhibit neuronal differentiation from stem cells and impede neuronal reprogramming (Su et al., 2014). Thus, proper regulation of SOX2 expression is critical for achieving successful neuroregeneration.

NG2 glial cells in the spinal cord also have neurogenic potential and can be reprogrammed through ectopic SOX2 expression, promoting functional neurogenesis and injury recovery (Wang and Zhang, 2018; Tai et al., 2021). Pericytes, which are cells that wrap around capillaries and regulate blood flow (Bergers and Song, 2005), and microglia, which are immune cells in the spinal cord, have also shown the potential to be reprogrammed into functional neurons in culture (Matsuda et al., 2019; Cakir et al., 2022; Feng et al., 2022). However, achieving sufficient neuronal reprogramming for repair without overly disrupting the physiological function of these cells is a crucial consideration. Achieving functional benefits through *in vivo* gene expression reprogramming can be challenging due to the numerous parameters that need to be optimized. These parameters may include the site(s) and timing for viral injection, the reprogramming factor(s) to use, the models of SCI, and the types of behavioral assays to perform. For instance, multiple injection sites may be necessary to reprogram a significant number of new neurons and achieve sufficient impact to facilitate functional recovery (Tai et al., 2021). Additionally, overexpression of TFs that only appear during development can activate unconventional signaling pathways in adult cells. In the future, research in *in vivo* gene expression reprogramming should aim to understand those TFs involved in the reprogramming process to enhance reprogramming efficiency.

### Future directions in neuronal regenerative medicine

Neuronal regenerative medicine is an advancing field that may have great potential for treating various neurological disorders, injuries, and degenerative conditions. To further enhance its effectiveness, researchers are exploring multiple approaches such as stem cell therapies, gene therapies, biomaterials, neurotrophic factors, and electrical stimulation. The integration of these approaches through combination therapies could maximize the regenerative potential of neuronal regenerative medicine. For example, (1) in the early stages after SCI, gene therapy could be used to achieve several goals: reduce the formation of glial scars and immune responses to create a more favorable microenvironment for neuronal regeneration; (2) ectopic expression of potential TFs, such as SOX2, specifically activates regenerative genes in particular cell types; (3) potential TF genes could be delivered using nanoparticles, a non-viral delivery method, to different cell types such as NG2+ cells; (4) using a nanoparticle approach, inhibitory genes such as CSPGs could be knocked down to reduce lesion size and other negative effects, such as reducing the CSPGs produced by NG2, while increasing fiber sprouting; and (5) use gene editing to modify histone proteins, which regulate gene activity and affect the gene-regulatory machinery, to open condensed chromatin and expose potential regenerative genes. For example, Weng et al. (2017) used CRISPR/dCas9 fused to the catalytic domain of the histone acetyltransferase p300 (dCas9-p300) to specifically target the promoter regions of regeneration-associated genes (RAGs) in cultured primary neurons. By increasing histone acetylation at these loci, the researchers successfully activated the expression of RAGs, which in turn enhanced axonal growth *in vitro*. This study demonstrates the potential of using the CRISPR/dCas9 system to modulate histone modifications and promote axonal regeneration. Future research could extend this approach to *in vivo* models of SCI or other nervous system injuries to evaluate its therapeutic potential for functional recovery.

Taken together, stem cell therapy could be combined with various gene therapies or biomaterials to further enhance the regeneration of damaged neurons. The future of neuronal regenerative medicine looks promising, with the potential for innovative therapies that could revolutionize the treatment of neurological conditions.

## Conclusions

In conclusion, while regenerative medicine offers hope for the development of new therapies for SCI, significant challenges still need to be overcome. Current strategies face limitations such as low cell survival rates and lack of integration into host tissue. Nonetheless, innovative approaches such as nanostructured biomaterials and *in vivo* gene expression reprogramming show promise in promoting neural regeneration and repair. Continued research into the molecular mechanisms underlying these approaches and optimizing parameters such as timing, dosing, and delivery methods could pave the way for more effective treatments in the future. With ongoing advancements in the field of regenerative medicine, there is hope for improved outcomes and better quality of life for individuals living with SCI.

Therefore, our opinion is that continuous efforts are needed to develop safe and effective therapies, and to better understand the mechanisms that regulate neuronal regeneration in the spinal cord. We provide some future directions for neuronal regenerative medicine: (a) highlight the importance of comparative studies of neuronal regeneration in different vertebrate species to gain insights into the underlying mechanisms and to identify potential targets for promoting regeneration in mammals; (b) to address the current limitations and challenges in promoting neuronal regeneration; and (c) combining two different gene therapy approaches may offer a potential way to promote neuronal regeneration and improve outcomes for patients with SCI.

## Author contributions

Both authors listed have made a substantial, direct, and intellectual contribution to the work and approved it for publication.
